# Evaluation of Postnatal Sedation in Full-Term Infants

**DOI:** 10.3390/brainsci9050114

**Published:** 2019-05-17

**Authors:** Jean Carmela Solodiuk, Russell William Jennings, Dusica Bajic

**Affiliations:** 1 Department of Anesthesiology, Critical Care and Pain Medicine, Boston Children’s Hospital, Boston, MA 02115, USA; jean.solodiuk@childrens.harvard.edu; 2 Esophageal Advance Treatment Center, Department of Surgery, Boston Children’s Hospital, Boston, MA 02115, USA; russell.jennings@childrens.harvard.edu; 3 Department of Surgery, Harvard Medical School, Boston, MA 02115, USA; 4 Department of Anaesthesia, Harvard Medical School, Boston, MA 02115, USA

**Keywords:** opioids, pain, pain management, pharmacotherapies, physical dependence, postoperative pain, midazolam, morphine, term, tolerance, weaning

## Abstract

Prolonged sedation in infants leads to a high incidence of physical dependence. We inquired: (1) “How long does it take to develop physical dependence to sedation in previously naïve full-term infants without known history of neurologic impairment?” and (2) “What is the relationship between length of sedation to length of weaning and hospital stay?”. The retrospective study included full-term patients over a period of one year that were <1 year of age and received opioids and benzodiazepines >72 hours. Quantification of fentanyl, morphine, and midazolam were compared among three time periods: <5 days, 5–30 days, and >30 days using *t*-test or one-way analysis of variance. Identified full-term infants were categorized into surgical (14/44) or medical (10/44) groups, while those with neurological involvement (20/44) were excluded. Physical dependence in full-term infants occurred following sedation ≥5 days. Infants with surgical disease received escalating doses of morphine and midazolam when administered >30 days. A positive association between length of sedation and weaning period was found for both respiratory (*p* < 0.01) and surgical disease (*p* = 0.012) groups, while length of sedation is related to hospital stay for the respiratory (*p* < 0.01) but not the surgical disease group (*p* = 0.1). Future pharmacological directions should lead to standardized sedation protocols and evaluate patient neurocognitive outcomes.

## 1. Introduction

As advances in pediatric critical care medicine have allowed for increased survival rates and management of more critically ill neonates and infants, the need for prolonged sedation and analgesia has increased. Indeed, pain management in neonates and children has been shown to decrease stress, morbidity, and mortality rates, and recovery times after surgery or disease [1]. Even without a source of surgical pain, critically ill infants and children receive sedation to reduce anxiety, agitation, and stress responses, and to facilitate ventilation [2]. Sedation is currently considered a component of safe and compassionate care of critically ill infants and children. However, prolonged sedation administration may result in the development of physical dependence to sedation medications [3,4,5]. Original studies with increased dosing and prolonged administration of opioids during neonatal and pediatric intensive care [4,6] demonstrated that neonates, infants, and children rapidly develop analgesic tolerance (defined as escalating drug dosage to achieve the same level of pain relief achieved initially) and physical dependence (defined as an adaptive state that develops from repeated drug administration and results in withdrawal upon cessation of drug use). In fact, opioid tolerance and dependence occurs more rapidly in younger age groups, develops more commonly during critical illness, and results more frequently from prolonged intravenous infusions of short-acting opioids [4].

Limited studies in premature infants reported short- [7,8] and long-term [9,10,11] negative neurodevelopmental outcomes following early postnatal opioid exposure. Since prolonged opioid sedation has been postulated to have possible negative long-term effects (see review [4]), current sedation management in intensive care units during mechanical ventilation uses a combination of opioids and benzodiazepines as a gold standard of sedation [12], despite the fact that morphine is considered safer than midazolam for prolonged administration [13]. Although prolonged sedation is defined as more than 72 hours of continuous sedation, since tolerance and physical dependence rarely occur before this time point [14,15], literature information is lacking regarding the length of sedation associated with development of physical dependence to sedative drugs (viz., opioids and benzodiazepines). To eliminate the added confound of prematurity, the main objective of our retrospective study was to identify the length of sedation in full-term infants (without any previously known neurological disease) leading to opioid and benzodiazepine dependence. Furthermore, we hypothesized full-term infants undergoing surgical treatment would be at a higher risk for prolonged sedation exposure and development of physical dependence when compared to those treated for medical illness and no surgery. It is our hope that this systematic retrospective investigation of sedation will lay the foundation for future clinical research into the immediate and long-term neurodevelopmental effects and neurocognitive outcomes of combined opioid and benzodiazepine administration in full-term infants. 

## 2. Materials and Methods

### 2.1. Patients

We conducted a retrospective cross sectional cohort study of full-term infants over a period of one year at a single institution. Ethical approval was obtained from Boston Children’s Hospital Institutional Review Board (Protocol Number IRB-P000007855) with classification as a no greater than minimal risk study. The study conformed to the standards set by the Declaration of Helsinki and Good Clinical Practice guidelines.

**Figure 1:**
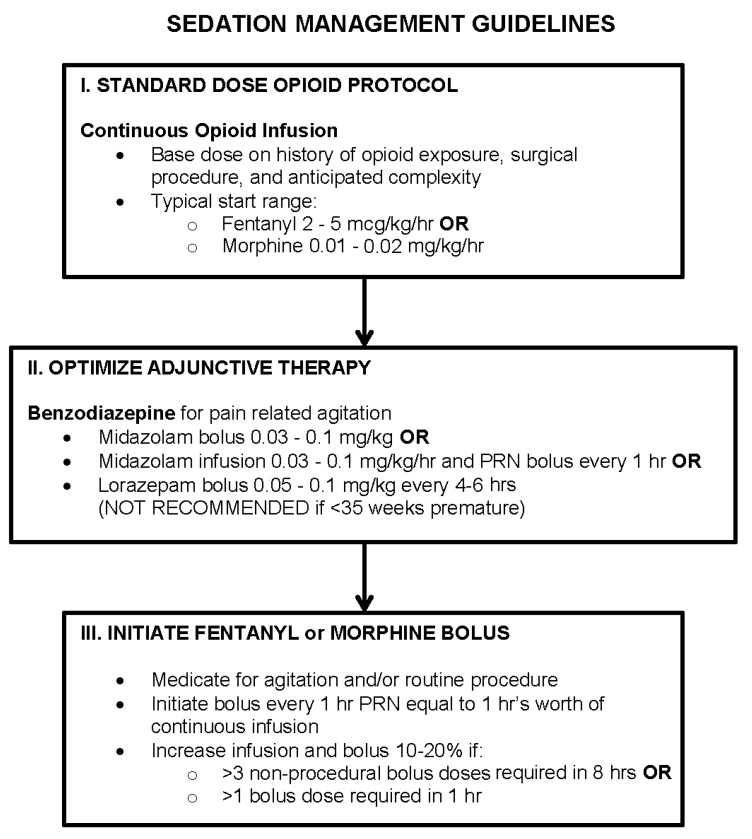
Prolonged Pain and Sedation Management Guidelines. Simplified summary of prolonged pain and sedation management is adapted from the Boston Children’s Hospital Patient Care Manual on Postoperative Pain Management. Continuous infusions of opioids and benzodiazepines are recommended for prolonged sedation. Guideline illustrates that sedation can be accomplished using various drugs and doses depending on individual patient’s needs, as well as physician and/or care service preference. There is no single, uniform sedation approach. Abbreviations: PRN, pro re nata (Latin) that means ‘as needed’; hr(s), hour(s).

We used three different search modes to maximize the number of subjects in the study and to increase the validity of data for the selected time period of one year. Specifically, we used: (1) the Informatics for Integrating Biology and the Bedside (i2b2) search platform (https://www.i2b2.org, a centralized repository of clinical data from a variety of Boston Children’s Hospital systems); (2) the Information Technology services at Department of Anesthesiology, Critical Care and Pain Medicine; and (3) the unit-specific patient search system from the neonatal intensive care unit (NICU). Search criteria were several: gestational age at birth (full-term, defined as birth between 37–42 weeks gestation), postnatal age at the time of admission (<12 months old), disease (pneumonia), treatment (intubation), and drugs (fentanyl, morphine, and midazolam). The search identified patients across all intensive care divisions that included the cardiac intensive care unit (CICU), medical intensive care unit (MICU), medical-surgical intensive care unit (MSICU), and NICU. Eligibility criteria were as follows: (1) full-term infants; (2) no previous exposure to opioids or benzodiazepines; (3) less than one year old at the time of treatment; and (4) endotracheal intubation and subsequent mechanical ventilation requiring concurrent (5) sedation using opioids and benzodiazepines (>72 hours) [15]. Cases of post-surgical intubation were included irrespective of intraoperative neuraxial/regional block application for pain control. Search modes inadequately identified infants that upon subsequent manual chart review had exclusionary (1) prenatal exposure to any sedative drugs (and/or drugs of abuse), (2) chromosomal abnormalities (e.g., Down’s syndrome), (3) prematurity (defined as birth at <37 weeks gestation), and/or (4) sedation for <3 days (since the search could not identify the length of treatment). Cases of non-sedated, non-invasive mechanical ventilation (e.g., via tracheostomy) were also excluded. All infants underwent sedation management as per Boston Children’s Hospital guidelines (Figure 1).

### 2.2. Chart Review

**Figure 2:**
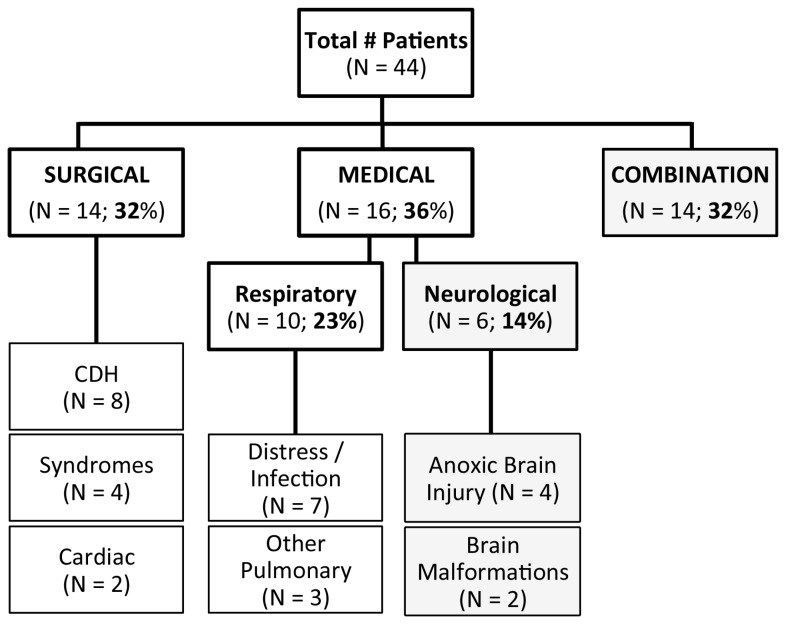
Identified disease profiles of patients undergoing prolonged sedation. Chart represents numerical summary of infants that met criteria for retrospective study over a search period of 1 year. Only full-term infants (without known chromosomal abnormalities) that received sedation for longer than 3 days in the first year of life were included. Figure summarizes disease profiles of all patients included in the study (*n* = 44). Approximately, one-third required surgical repair (*n* = 14; 32%), another third underwent medical treatment (either due to respiratory (*n* = 10; 23%) or neurological disease (*n* = 6; 14%)), and the remaining third had a combination of either surgical or medical disease that also included neurological involvement (*n* = 14; 32%). Infants undergoing major surgery (*n* = 14) were diagnosed with various diseases such as congenital diaphragmatic hernia (CDH), cardiac anomalies, or syndromes (diseases with more than one identifying feature or symptom). Infants with respiratory diseases (*n* = 10) were diagnosed with respiratory distress/infection or other (e.g., pulmonary hypertension or hemorrhage).

All the clinical data were obtained from an electronic medical record, Powerchart^R^ (Cerner, London, UK). Specifically, patients meeting inclusion criteria were categorized by primary diagnoses into those with (1) congenital disease, deformity, birth defect, or anomaly requiring surgical repair (cardiac and/or gastrointestinal systems; *n* = 14), or (2) primary respiratory disease (*n* = 10). Patients with neurological disease (e.g., anoxic/hypoxic-ischemic brain injury; Figure 2) with (*n* = 14) or without (*n* = 6) combinations of other diagnoses were excluded from subsequent analysis. In other words, to eliminate the bias of central nervous system disease, only charts from full-term infants without any known neurological injury/disease were analyzed for pharmacological sedation management. 

According to the retrospective study design [16,17,18], baseline state (underlying disease), the intervention (intubation requiring sedation), and the outcome (development of drug dependence) were obtained from existing clinical documentation that was recorded as part of the clinical treatment. Sedation was defined by period of intubation, while weaning was defined as period following extubation that required withdrawal management to prevent signs of physical dependence. Weaning of medications following extubation is not to be confused with patterns of sedation weaning (i.e., ‘lifting the sedation’) of critically ill children prior to extubation. This study did not analyze potential symptoms of withdrawal or withdrawal prevention management (weaning treatment). For more details on the latter subject, please see a recent review [19] since it is beyond the scope of this manuscript. We confirmed that administration of opioids and benzodiazepines was indicated specifically for weaning management as per primary team and/or pain service consults notes. In other words, physically dependent patients continued to receive sedative drugs (either opioids or benzodiazepines) following extubation to minimize expression of signs and symptoms of withdrawal. 

Quantitative analysis included the following end-points: (1) length of hospitalization (days); (2) length of intubation requiring sedation (days); (3) occurrence (yes/no) and length of drug weaning treatment (days) as a marker of physical dependence; (4) type of all the drugs used for sedation and weaning (including adjuncts); and (5) total average daily dose (unit/kg/day ± standard deviation (SD)) of drugs most frequently used for sedation only (opioids and benzodiazepines). To effectively compare the amount of selected medication administered to infants of varying weights during the period of sedation, average daily dose (unit/kg/day) was calculated using the following formulas:

(1) $$\mathrm{Infusion}\;\left(\mathrm{unit}\right)=\mathrm{dose}\;\left(\frac{unit}{kg}∕\mathrm{hour}\right)\times \;\mathrm{weight}\;\left(\mathrm{kg}\right)\times \;\mathrm{time}\;\left(\mathrm{hours}\right)$$

(2) $$Dose\;\left(\frac{unit}{kg}\right)=\frac{infusion\;\left(unit\right)+boluses\;\left(unit\right)\;during\;sedation}{average\;weight\;during\;sedation\;\left(kg\right)}$$

(3) $$Average\;Daily\;Dose\;\left(\frac{unit}{kg}/day\right)=\frac{average\;dose\;\left(unit/kg\right)}{sedation\;length\;\left(days\right)}$$

### 2.3. Statistical Analysis

Data such as treatment duration (days) and average daily dose (unit/kg/day) were individually analyzed and presented by using mean (± SD). We used either a Student’s *t*-test or a one-way analysis of variance with Tukey’s Honestly Significant Difference test to identify differences in average daily dose. Pearson’s correlation coefficient was used to measure the linear relationships between length of sedation (days) to either length of weaning or hospital stay (days). Statistical analyses were performed using VassarStats, a statistical computation site (http://vassarstats.net), while correlation coefficient calculations were done using Microsoft Excel^R^ (v.14.6.8, Microsoft Corp., Redmond, WA, USA). Statistical significance was assessed at the α < 0.05 level. Correlation coefficient of determination (*r*^2^) approaching 1 measured how close the data fit to the correlation line.

## 3. Results

### 3.1. Patient Diagnoses

The retrospective chart review included 221 charts, of which only 44 met the inclusion criteria. As illustrated in Figure 2, primary diagnoses were categorized into three groups: (1) surgical (14/44), (2) medical (either isolated respiratory (10/44) or neurological involvement (6/44)), and (3) combination of diseases that also included neurological involvement (14/44). Infants with sole neurological conditions had diagnoses that included either brain malformations (2/6) or anoxic brain injury (4/6). In total, neurological involvement was present in almost half of identified critically ill full-term infants that underwent sedation >3 days (20/44). To minimize bias of neurological disease, subsequent detailed quantitative analysis of sedation included full-term infants without any known neurological involvement (*n* = 24; Figure 2). All infants in the surgical group underwent repair of congenital anomalies (that excluded known/identified abnormalities of the central nervous system) and had a variety of diagnoses that included: (1) gastrointestinal anomalies such as congenital diaphragmatic hernia (CDH; 8/14); (2) syndromes/multiple anomalies (4/14) such as CHARGE (coloboma of the eye, heart defects, atresia of the choanae, retardation of growth and/or development, genital and/or urinary abnormalities, ear abnormalities and deafness) or VACTERL (vertebral defects, anal atresia, cardiac defects, trachea-esophageal abnormalities, renal abnormalities, limb abnormalities); and (3) cardiac anomalies such as aortic coarctation and heart failure (2/14). Infants in the respiratory disease group had diagnoses of respiratory distress/infection (7/10; e.g., pneumonia), or other pulmonary diseases such as idiopathic pulmonary hypertension or hemorrhage (3/10).

### 3.2. Sedation Drugs

Only full-term infants undergoing surgical repair or treatment of respiratory disease were subsequently assessed for the extent of sedation. Summary of demographic characteristics for full-term infants with respiratory and surgical disease is listed in Table 1.

Opioids (fentanyl and/or morphine) and midazolam (a benzodiazepine) were the main drugs used for sedation. In addition to being administered as infusions, they were also given as intermittent boluses to deepen sedation as per Boston Children’s Hospital Sedation Guidelines (Figure 1). In some cases, only fentanyl (*n* = 4/6 respiratory group; *n* = 2/11 surgical group) or morphine (*n* = 4/6 respiratory group; *n* = 3/12 surgical group) was administered. In other cases, these two opioids were administered together (*n* = 2/10 respiratory group; *n* = 9/14 surgical group). Additional drugs included other opioids (methadone), benzodiazepines (lorazepam), alpha2-adrenergic agonists (dexmedetomidine, clonidine), and/or medications that are considered general anesthetics (propofol, ketamine). Dexmedetomidine and ketamine were used as infusions to supplement sedation with opioids and benzodiazepines on an individual basis. Furthermore, longer-acting methadone and lorazepam were administered only as intravenous boluses, while clonidine was administered as either an intravenous bolus or a skin patch. All of these additional medications were selected as per individual physician’s preference with a goal to decrease overall administration of primary sedation drugs (fentanyl and/or morphine, together with midazolam). Detailed summary of sedation drugs administered to individual patients with respiratory and surgical disease are summarized in Table 2 and Table 3, respectively.

### 3.3. Sedation Management

**Figure 3:**
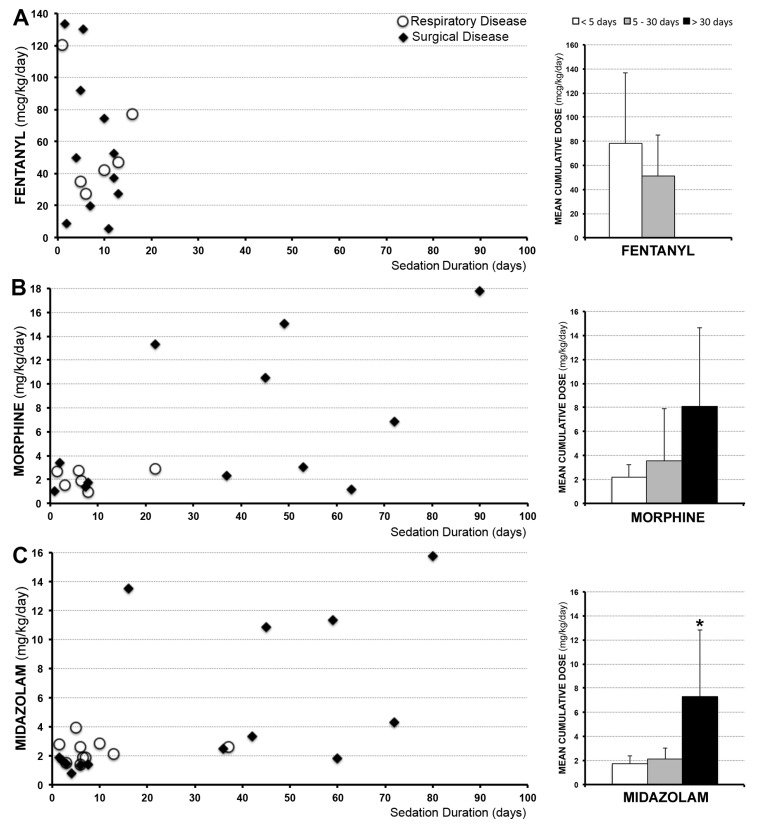
Individual average daily dose of drugs used for sedation. Graphs on the left illustrate individual average daily dose received (unit/kg/day) of medications (fentanyl (**A**), morphine (**B**), and midazolam (**C**)) administered to full-term infants treated for either respiratory (*n* = 10; white circles) or surgical (*n* = 14; black diamonds) disease during a single hospital admission (first or only hospital admission). Patients were selected over a period of one year from a single institution. Individual infants received fentanyl ((**A**); *n* = 17) and/or morphine ((**B**); *n* = 18); not all infants received both. Every infant received midazolam ((**C**); *n* = 24). Note that fentanyl was not administered for longer than 2 weeks. Bar graphs on the right show mean of the individual average daily dose received for both groups across three different arbitrary time periods: <5 days (white), 5–30 days (grey), and >30 days (black). There was no significant difference in mean values for fentanyl (*t* = 1.16, *p* = 0.13) or morphine (F(2,15) = 2.28, *p* = 0.14) among different time periods. The mean individual average daily dose for midazolam was significantly higher when used for sedation >30 days (F(2,21) = 6.91, *p* <0.01). Asterisk (*) indicates significant difference.

Considering that opioids (fentanyl and/or morphine) were used in combination with a benzodiazepine (midazolam) in all patients requiring sedation >3 days (Table 1 and Table 2), we quantified treatment length and average daily drug doses (unit/kg/day) of each of these three drugs (fentanyl, morphine, and midazolam). Although specific drug administration varied between patients, sedation with opioids and benzodiazepines for ≥5 days was associated with development of physical dependence requiring subsequent opioid and benzodiazepine weaning (Table 2 and Table 3). Quantification of individual drug administration for patients with respiratory (*n* = 10) and surgical disease (*n* = 14) is shown in Figure 3 (left columns). Variability in average daily drug doses for sedation was noted among individual patients in both groups. As a result, mean values for both groups (*n* = 24) were calculated for the following arbitrary time periods: <5 days, 5–30 days, and >30 days (Figure 3; right columns). No infants received fentanyl for >2 weeks (Figure 3A). Although the mean average daily dose for fentanyl was lower if administered ≥5 days (51.31 ± 34.04) in comparison to first 4 days (77.98 ± 58.99), no significant difference was noted (*t* = 1.16, *p* = 0.13). Sedation with morphine and midazolam >30 days was associated with higher average daily doses despite co-administration of longer acting opioids, benzodiazepines, and/or sedative adjuvants (Table 2 and Table 3). However, we report significant increase for midazolam (7.33 ± 5.46; F(2,21) = 6.91, *p* <0.01), but not morphine (8.09 ± 6.54; F(2,15) = 2.28, *p* = 0.14). Sedation >30 days was chiefly identified in infants undergoing surgical disease repair, with the exception of one patient with respiratory disease who was treated for idiopathic pulmonary hypertension.

### 3.4. Time Correlations

**Figure 4:**
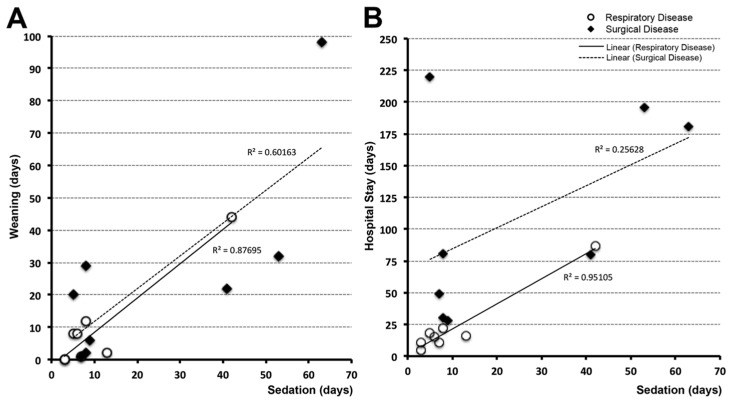
Correlation of length of sedation with length of weaning and hospital stay. Panel (**A**) demonstrates linear correlation between length of weaning (days) and length of sedation (days) for respiratory (*n* = 8; white circles) and surgical (*n* = 8; black diamonds) disease groups. In other words, longer periods of sedation were associated with increased length of weaning, irrespective of disease (respiratory (*r*^2^ = 0.88, *p* <0.01); surgical (*r*^2^ = 0.60, *p* = 0.01)). Panel (**B)** demonstrates linear correlation between length of sedation and length of hospital stay for the respiratory disease group (*r*^2^ = 0.95, *p* ≤ 0.01), but not the surgical group (*r*^2^ = 0.26, *p* = 0.1).

Correlation analyses explored the association between length of sedation and other variables such as length of drug weaning (Figure 4A) and length of hospital stay (Figure 4B). Surgical and respiratory disease groups were adjusted as follows: 2/10 patients from the respiratory group were excluded due to transfer before completion of weaning; 6/14 patients from the surgical group were excluded either due to either transfer before completion of weaning (*n* = 1), medical/surgical complications during weaning treatment (*n* = 3), or death (*n* = 2). We report positive linear relationship between length of sedation and weaning for both the respiratory (*n* = 8, *r*^2^ = 0.88, *p* <0.01) and surgical groups (*n* = 8, *r*^2^ = 0.60, *p* = 0.01) (Figure 4A). The positive association between length of sedation and length of hospital stay was significant for the respiratory disease group (*n* = 8, *r*^2^ = 0.95, *p* <0.01) but not the surgical group (*n* = 8, *r*^2^ = 0.26, *p* = 0.1; Figure 4B). Described linear relationships do not provide any causative information.

## 4. Discussion

In the current retrospective report, we show that physical dependence to opioids and benzodiazepines in full-term infants develops following sedation ≥5 days irrespective of the primary disease (respiratory vs. surgical). We also show that full-term infants with surgical disease receive longer sedation over the course of clinical treatment. Significant linear correlations confirm association between length of sedation and length of drug weaning for both patient populations. In contrast, length of sedation does not appear to be the sole factor contributing to length of hospitalization for surgical group. 

### 4.1. Limitations of the Retrospective Chart Review

We followed recommended methodology of retrospective investigative technique [20] to assess its major benefits and minimize limitations. In accordance with retrospective analyses, our study included data originally collected for reasons other than research [16,21]. As such, retrospective analysis is vulnerable to incomplete or missing documentation, poorly recorded or absent chart information (e.g., apparent lack of sedation scales), as well as difficulty in identification of desired patients (e.g., possible underestimation of total number of patients).

#### 4.1.1. Study Size

Despite using three different search modes (see Section 2.1) for the most common method of sampling (‘convenience sampling’ over a specific time frame [17]), a large number of patient candidates needed to be excluded since search criteria did not distinguish in cases of poorly documented gestational age (e.g., <37 weeks), presence of chromosomal abnormalities (e.g., Down’s syndrome), or length of sedation (e.g., one vs. several days). Furthermore, to minimize bias of neurological disease, approximately half of the identified candidates were further excluded due to known pre-existing neurologic abnormalities (e.g., anoxic/hypoxic-ischemic brain injury; Figure 2). A sample size of 10 cases (charts) per variable is sufficient to obtain results that are likely to be both true and clinically useful [22]. While the literature generally holds ten events per predictor as an accepted norm [22,23,24], others have suggested that it is acceptable to have a minimum of seven or five events per predictor [25]. Although our final sample numbers are small, they are within the range accepted in the literature. Due to unbalanced nature of the sample of total 24 subjects analyzed (i.e., differing number of patients with respiratory vs. surgical disease), it is possible that query might not be sufficiently powered.

#### 4.1.2. Withdrawal Evaluation

Our analysis relied only on physician’s notes for confirmation of withdrawal management. In the absence of quantification of any objective findings (viz., physiological signs of dependence or withdrawal scores), bias may be introduced regarding conclusion that physical dependence occurs ≥5 days of sedation. Therefore, future follow up studies should more closely look into evaluation of withdrawal scores [26,27] to strengthen currently presented data.

### 4.2. Sedation of Full-Term Infants

Previous limited literature states that tolerance and physical dependence rarely occur before 72 hours of continuous sedation [14,15]. Our report demonstrates that sedation ≥5 days is associated with physical dependence irrespective of complexity of the underlying disease. Presented data also show that full-term infants undergoing surgical treatment are at higher risk for longer sedation in comparison to those with medical illness. As illustrated in Figure 3, fentanyl was not administered longer than 2 weeks. Being more lipophilic, fentanyl is known to be associated with faster onset of opioid tolerance [4,6,28] and is commonly replaced by longer acting opioids and sedation adjuvants during clinical management. Indeed, the most commonly used sedation regimen for intubated children is a combination of opioids and benzodiazepines (72%) [29]. As shown in Table 2 and Table 3, additional longer-acting opioids (viz., methadone) and benzodiazepines (viz., lorazepam), together with different adjuvants (viz., clonidine, ketamine, etc.) were administered with a goal to minimize dosing escalations of morphine and midazolam as primary sedative drugs. Despite such efforts, our findings of escalating administration of sedative drugs (viz., morphine and midazolam) during prolonged sedation align with previously published results by Best et al. [30]. Similar to our single institution study, the majority of patients in their study (from 22 institutions within the United States) also received fentanyl and/or morphine together with midazolam as their primary sedative drugs. Although their study population was large (145 patients vs. 24 in our study), the age range was large (2 weeks to 17 years old vs. full-term infants <12 months old in our study) and authors did not specify whether their children were born full-term or premature. The recent multicenter MOTIF (Measuring Opioid Tolerance Induced by Fentanyl (or morphine)) study [31] reported that doubling of the daily opioid dose was more likely to occur following opioid infusion for 7 days or longer, or post co-therapy with midazolam. A few international studies [29,32,33] reported significant variation in clinical approaches of sedation practices in pediatric critical care, which is similarly reflected in our data (Table 2 and Table 3). Future quantitative studies should investigate nature of the surgical critical care requiring prolonged sedation (e.g., impact of complications, need for additional procedures and/or surgical revisions).

### 4.3. Sequalae of Prolonged Postnatal Opioid and Benzodiazepine Exposure

A growing body of evidence supports the notion that long-lasting neurobehavioral and/or cognitive disturbances may be a consequence of prenatal opioid exposure [34,35]. However, studies of long-term safety or neurobehavioral and neurocognitive outcomes of postnatal exposure to prolonged opioids and/or benzodiazepines are limited and confined to investigations in premature infants. 

#### 4.3.1. Opioids

Some studies show that prolonged sedation and/or analgesia is not associated with a poor 5-year neurological outcome after adjustment for the propensity score [36,37]. These authors even went on to conclude that continuous morphine infusion of 10 mcg/kg/h during the neonatal period does not harm general functioning and may even have a positive influence on executive functions at 8 to 9 years. Low-dose morphine analgesia in premature infants is associated with early alterations in cerebral structure and short-term neurobehavioral problems that did not persist into childhood [8]. In contrast, morphine infusions administered during the neonatal period for sedation of premature infants during mechanical ventilation led to long-term neurodevelopmental delay, as well as neurocognitive and motor impairment at the age of 5 years [10,38]. Similarly, results from the recent NEOPAIN (Neurological Outcomes and Preemptive Analgesia in Neonate) trial [11] in premature infants strongly suggest long-lasting negative effects. Specifically, body weight and head circumference remained diminished at 5 to 7 years of age in the preemptive morphine-treated group as compared to the group treated with placebo. The former premature infants in the experimental group also had more social problems and exhibited increased latencies to choice responses in the short-term memory task [11]. Reports of increased pain sensitivity [39], altered anxiety and stress responses, metabolic changes [40], and potential memory changes (through amygdalo-frontal circuits) [11] have also been reported. These changes may also reflect an overall hypersensitivity and maladaptation to adverse conditions [41] in premature infants. It is however, well established that prematurity alone is associated with numerous neurological sequelae [42], including high risk for cerebral injuries such as hypoxia–ischemia events, stroke, and periventricular leukomalacia [43] implicating prematurity as a significant confounding factor in evaluating neurodevelopment.

#### 4.3.2. Benzodiazepines

Similar to opioid research, longitudinal studies focused on benzodiazepine exposure are lacking. Recently, one study found that prior exposure to midazolam was associated with impaired hippocampal growth and poorer neurodevelopmental outcomes in infants at an 18-month follow up [44]. In a study of one-year-old infants who previously underwent cardiac surgery (arterial switch operation), total midazolam dose was shown to adversely affect neurodevelopmental outcome [45,46]. However, when assessing the entire study population, the Bayley Scales of Infant and Toddler Development composite score was within the normal range for given population [45,46], and additional longitudinal follow-up of this cardiac cohort has not occurred. Another study by Guerra et al. found a small, but statistically significant association between days of sedation and lower performance intelligence quotient, as well as between benzodiazepine cumulative dose and lower Visual Motor Integration assessment [47,48]. While a growing body of literature implicates benzodiazepine exposure as a causal factor in poor developmental outcomes, selected population of cardiac patients introduces the major confound of surgery with cardiopulmonary bypass and associated stress, precluding assertions at this time.

#### 4.3.3. Future Studies

Despite the common clinical pairing of opioids with benzodiazepines [29], no studies to date have evaluated the effects of their simultaneous administration on longitudinal neurodevelopment. Our recent work showed incidental brain magnetic resonance imaging findings and quantitative evidence of smaller total brain volumes in both premature and full-term infants following complex perioperative and critical care for thoracic noncardiac congenital anomalies (viz., long-gap esophageal atresia requiring the Foker process) that involved administration of opioids and benzodiazepines for sedation [49]. Similarly, recent work from group in the Netherlands demonstrated that both premature and full-term infants undergoing surgical repair of noncardiac congenital anomalies are at risk of brain injury [50], potentially accounting for the neurodevelopmental delay frequently observed in this population [51]. Our lack of clear understanding regarding potential negative neurobehavioral outcomes in full-term children exposed to prolonged sedation in infancy should be investigated in future studies that minimize potential biases (e.g., prematurity and surgery involving cardiopulmonary bypass). Importantly, the present manuscript opens the path for such investigations by shedding light on two specific populations of full-term infants: (1) those with respiratory disease (without confounds of surgery and anesthesia) that are treated within 2–3 weeks, and (2) select infants with noncardiac anomalies requiring thoracic surgery (without confound of cardiopulmonary bypass or extracorporeal membrane oxygenation) that are at need of prolonged sedation >30 days.

## 5. Conclusions

Sedation and analgesia are important components in the management of intubated and mechanically ventilated patients, as patient comfort and safety are the central goals. Our retrospective study analyzed length of sedation and weaning suggesting that physical dependence to sedation drugs (opioids and benzodiazepines) occurs with sedation ≥5 days in full-term infants treated for either respiratory or surgical disease. Given our limited understanding of the immediate and long-term effects of prolonged sedation on brain development, future research should include groups of patients delineated in this report, so as to explore the impact of opioids and benzodiazepines on neurocognitive outcomes in the absence of prematurity and cardiopulmonary bypass confounds.

## Figures and Tables

**Table 1:** Demographic Characteristics. Summary of demographic characteristics of full-term patients in the surgical and respiratory disease groups that were included in the comprehensive data analysis. Such analysis excluded any full-term infants with known neurological disease.

	Disease Category	
Characteristics	Surgical (*N* = 14)	Respiratory (*N* = 10)
Male Gender—*n* (%)	11 (79%)	5 (50%)
Gestational age at birth (wks) ± SD	39.1 ± 1.6	39.3 ± 1.1
Multiple births— *n* (%)	0	1 (10%)
Birth by C/S— *n* (%)	6 (43%)	2 (20%)

Abbreviations: C/S, cesarean section; SD, standard deviation; wks, weeks; %, percent.

**Table 2:** Patients undergoing respiratory disease sedation treatment. Patients with respiratory diseases are arranged according to disease category (respiratory distress/infection vs. other) and length of sedation. Data includes sedation treatment and weaning for each infant from a single hospital admission at Boston Children’s Hospital; infants did not require multiple hospital admissions. Sedation period coincided with intubation. All patients were sedated with a combination of opioids (fentanyl and/or morphine) and benzodiazepine (midazolam), irrespective of intensive care location. Average daily dose (unit/kg/day) is defined as the average daily total dose of each drug received both as an infusion and boluses. Other additional pain and sedation medications such as clonidine, dexmedetomidine, and lorazepam (administered in the form of boluses, unless otherwise stated) were administered according to physician and/or care service preference. Patients with sedation <5 days (*n* = 2/10) did not require drug weaning following extubation.

#	Sedation/Intubation (days)	LOS	Location	Drugs	Average Dose (unit/kg/day)	Other Sedatives	Weaning
**Respiratory Distress/Infection**							
1	3	4	MSICU	Morphine	2.67 mg	None	No
				Midazolam	2.80 mg		
2	3	10	MSICU	Morphine	1.56 mg	None	No
				Midazolam	1.52 mg		
3	5	17	NICU	Fentanyl	35.21 mcg	Lorazepam	Yes
				Midazolam	3.96 mg		
4	7	8	MSICU	Morphine	1.92 mg	Lorazepam	Yes
				Midazolam	1.88 mg		
5	7	10	MSICU	Morphine	2.72 mg	Lorazepam	Yes
				Midazolam	2.63 mg		
6	13	15	NICU	Fentanyl	46.86 mcg	Lorazepam	Yes
				Midazolam	2.11 mg		
7	14	14	MSICU	Fentanyl	27.20 mcg	Clonidine, dexmedetomidine (infusion), lorazepam	Yes
				Midazolam	1.37 mg		
**Other Pulmonary Disease**							
8	9	21	MSICU	Fentanyl	120.14 mcg	Lorazepam	Yes
				Morphine	0.95 mg		
				Midazolam	1.88 mg		
9	10	10	NICU	Fentanyl	42.01 mcg	None	Yes
				Midazolam	2.82 mg		
10	37	86	MSICU	Fentanyl	77.20 mcg	Clonidine (patch)	Yes
				Morphine	2.91 mg		
				Midazolam	2.60 mg		

Abbreviations: LOS, length of stay; MSICU, medical-surgical intensive care unit; NICU, neonatal intensive care unit.

**Table 3:** Sedation management of patients undergoing surgical repair. Patients undergoing surgical treatment are arranged according to disease category (syndromes/multiple anomalies, cardiac, and congenital diaphragmatic hernia) and length of sedation. Presented data include sedation treatment and weaning for each infant from a single hospital admission (first, or only, hospital admission) at Boston Children’s Hospital. Patients numbered 1, 2, 5, 7, and 14 required multiple hospital admissions or multiple sedation treatments. Sedation period coincided with intubation. All patients were sedated with a combination of opioids (fentanyl and/or morphine) and benzodiazepines (midazolam), irrespective of intensive care location. Average daily dose (unit/kg/day), defined as the total dose of each drug received both as an infusion and boluses, increased with the length of sedation. Other additional pain and sedation medications administered included clonidine, dexmedetomidine, ketamine, lorazepam, methadone, and oxycodone (administered in the form of boluses, unless otherwise stated). Patient with sedation <5 days did not require weaning following extubation. Asterisks (*) indicate patients were deceased at the time of screening.

#	Sedation/Intubation (days)	LOS	Location	Drugs	Average Dose (unit/kg/day)	Other Sedatives	Weaning
**Syndromes**							
1	4	170	MSICU	Fentanyl	27.24 mcg	None	No
				Midazolam	1.47 mg		
2	5	219	MSICU	Fentanyl	91.96 mcg	Oxycodone (liquid)	Yes
				Morphine	1.03 mg		
				Midazolam	0.81 mg		
3	7	48	NICU	Fentanyl	19.71 mcg	None	Yes
				Midazolam	1.70 mg		
4	8	80	NICU	Morphine	1.38 mg	None	Yes
				Midazolam	1.41 mg		
**Cardiac**							
5	8	164	CICU	Fentanyl	133.25 mcg	Dexmedetomidine (infusion), lorazepam (liquid and bolus), methadone (liquid and bolus)	Yes
				Morphine	1.73 mg		
				Midazolam	1.31 mg		
6	9	27	CICU	Fentanyl	50.00 mcg	None	Yes
				Morphine	3.40 mg		
				Midazolam	1.90 mg		
**Congenital Diaphragmatic Hernia**							
7	27	115	MICU	Fentanyl	130.12 mcg	Dexmedetomidine (infusion), ketamine, lorazepam, methadone	Yes
				Morphine	13.29 mg		
				Midazolam	13.53 mg		
8	41	105	MSICU	Morphine	2.29 mg	Clonidine (patch and liquid), lorazepam (liquid and bolus), methadone (liquid)	Yes
				Midazolam	2.50 mg		
9*	45	45	MSICU	Fentanyl	37.15 mcg	Dexmedetomidine (infusion), lorazepam	Yes
				Morphine	10.51 mg		
				Midazolam	10.84 mg		
10	53	53	MSICU	Morphine	3.05 mg	Clonidine (patch), dexmedetomidine (infusion), ketamine, lorazepam (liquid and bolus)	Yes
				Midazolam	3.33 mg		
11	63	148	MSICU	Fentanyl	74.54 mcg	Clonidine (patch and liquid), dexmedetomidine (infusion), ketamine, lorazepam (liquid)	Yes
				Morphine	15.06 mg		
				Midazolam	11.32 mg		
12	64	91	MSICU	Fentanyl	8.53 mcg	Clonidine (patch), lorazepam (liquid)	Yes
				Morphine	1.16 mg		
				Midazolam	1.83 mg		
13 *	70	71	MSICU	Fentanyl	52.44 mcg	None	Yes
				Morphine	6.82 mg		
				Midazolam	4.30 mg		
14	90	248	MSICU	Fentanyl	5.40 mcg	Dexmedetomidine (infusion), lorazepam, methadone	Yes
				Morphine	17.77 mg		
				Midazolam	15.75 mg		

Abbreviations: LOS, length of stay; CICU, cardiac intensive care unit; MICU, medical intensive care unit; MSICU, medical-surgical intensive care unit; NICU, neonatal intensive care unit.

## Abbreviations

CDH	congenital diaphragmatic hernia
CICU	cardiac intensive care unit
i2b2	Informatics for Integrating Biology and the Bedside
MICU	medical intensive care unit
NEOPAN	Neurological Outcomes and Preemptive Analgesia in Neonates
NICU	neonatal intensive care unit
MOTIF	Measuring Opioid Tolerance Induced by Fentanyl (or morphine)
